# A MIXED METHODS SYSTEMATIC REVIEW OF COMMUNITY-BASED GROUPS FOR SOCIAL ISOLATION AND LONELINESS IN OLDER PEOPLE

**DOI:** 10.1093/geroni/igz038.3147

**Published:** 2019-11-08

**Authors:** Brenda A Hayanga, Dylan Kneale, Ann Phoenix

**Affiliations:** 1 University College London, England, United Kingdom; 2 The Evidence for Policy and Practice Information and Co-ordinating Centre, Institute of Education, University College London, England, United Kingdom; 3 Thomas Coram Research Unit, Institute of Education, University College London, England, United Kingdom

## Abstract

In the UK, many older people from minoritised ethnic groups are vulnerable to social isolation and loneliness. Yet, we know little about which interventions are effective for them. With existing systematic reviews of social isolation and loneliness lacking a theory-based framework of their life-course experiences, we set out to address this gap. This review aims to explore the effectiveness and suitability of community-based group interventions (CBGIs) for social isolation and loneliness in older people. The decision to focus on CBGIs was based on findings from an exploratory study of the friendship networks of older people and narrative interviews with older minoritised people living in the UK. The findings suggested that community groups of shared interests/backgrounds were protective of social isolation and loneliness. To address the objectives, we searched for randomised controlled trials and process evaluations of CBGIs published in English, which included older people living in countries with membership to the Organisation for Economic Co-operation and Development. We identified 4791 studies, 36 of which were eligible for inclusion. In this poster, we present the preliminary findings of this mixed-methods systematic review, which seeks to not only assess whether CBGIs are effective but also to understand the underlying processes that make interventions (in)effective. As this review is guided by findings from two exploratory studies with older people from minoritised ethnic groups, it takes into account their life-course experiences. It is the results of reviews such as this that can produce generalisable findings which are directly applicable to policy.

